# Supplementary data for genome-wide DNA methylation and gene expression analysis of PBMCs in patients with metabolic syndrome

**DOI:** 10.1016/j.dib.2022.108183

**Published:** 2022-04-13

**Authors:** Su-Jin Baek, Siwoo Lee, Hee-Jeong Jin

**Affiliations:** KM Data Division, Korea Institute of Oriental Medicine, 1672 Yuseong-daero, Yuseong-gu, Daejeon, 34054, Republic of Korea

**Keywords:** Biomarker, Multi-omics, Epigenome, Transcriptome, Correlation

## Abstract

This supplementary data supports the research article ‘Genome-wide DNA methylation profiling reveals candidate biomarkers and probable molecular mechanism of metabolic syndrome’ (Baek et al., in press). To obtain these data, 32 participants with metabolic syndrome (MetS) were enrolled in the associated study. We collected peripheral blood mononuclear cells (PBMCs) from 11 patients with MetS and nine controls and compared genome-wide gene expression and DNA methylation signatures. The remaining 12 participants were used for the experimental validation of the candidate groups. We provide the raw, analyzed, and filtered genome-wide DNA methylation data, obtained using the Infinium Human MethylationEPIC BeadChIP array, and whole transcriptome sequencing data (accession number GSE181647). We list the differentially expressed and differentially methylated genes and their biological functions. These data can serve as a basis for screening appropriate epigenetic biomarkers for MetS.

## Specifications Table

**Table:** 

Subject	Biological sciences
Specific subject area	Omics: Epigenome, Transcriptome
Type of data	RNA sequence data, Human Methylation EPIC BeadChIP array data, and tables
How the data were acquired	We isolated genomic DNA from peripheral blood mononuclear cells (PBMCs; QIAamp DNA MiniKit, Qiagen). Bisulfite-converted genomic DNA was used for hybridization with Illumina's Infinium MethylationEPIC BeadChIP. We quantified methylation using the average normalized beta value via ChAMP. We isolated total RNA from PBMCs using TRIzol RNA Isolation Reagents (Thermo Fisher Scientific). Genome-wide methylation and gene expression were analyzed using a Human MethylationEPIC BeadChIP Kit (Illumina), and total RNA-sequencing was performed using the MGISEQ-2000 platform (MGI-Tech).
Data format	Raw (Fastq, idat), analyzed, and filtered data.
Description of data collection	The data were from 32 participants who were enrolled in the associated study. Of these, 20 were used to produce the RNA sequence and methylation array data for analysis, and the remaining 12 participants were used to validate the candidate.
Data source location	Korea Institute of Oriental Medicine, Daejeon, Republic of Korea
Data accessibility	The raw and processed MethylationEPIC BeadChIP and RNA-seq data have been deposited in the NCBI Gene Expression Omnibus (GEO), accession number GSE181647. Direct URL to data: https://www.ncbi.nlm.nih.gov/geo/query/acc.cgi?acc=GSE181647. Supplementary data link: https://data.mendeley.com/datasets/wgsjz42pg8/2
Related research article	Baek, S.J., Ban, H.J., Park, S.M., Kim, S.Y., Lee, S. and Jin, H.J., Genome-wide DNA methylation profiling reveals candidate biomarkers and probable molecular mechanism of metabolic syndrome. Genes & Diseases. (2022). https://doi.org/10.1016/j.gendis.2021.12.010

## Value of the Data

• These genome-wide DNA methylation and gene expression data can provide insight into metabolic syndrome, for review and meta-analysis purposes.
• The data will benefit researchers studying the cost-effectiveness of metabolic syndrome biomarkers.
• The data can be used in future studies of the mechanisms and targets whereby DNA methylation regulates gene expression in metabolic syndrome.

## 1 Data Description

These supplementary data, associated with Baek et al.[1], comprise the raw data and the analyzed and filtered epigenetic biomarker data for patients with MetS and controls. Raw data (GEO accession: GSE181647) include RNA sequence (fastq) and methylation array (idat) data for 20 participants. Patient characteristics are listed in Table S1. Table S2 lists the differentially methylated probes (DMPs), comparing patients with MetS and controls. Table S3 describes the functional enrichment of genes under hyper- and hypomethylation conditions. Table S4 presents the differentially expressed genes (DEGs). Table S5 describes the functional enrichment of the DEGs, using the DisGeNET gene sets. Table S6 lists the genes with significant correlations between their expression and DNA methylation. Table S7 describes the integrated functional network genes and pathways.

List of Tables

Table S1. Demographics and characteristics of study population

Table S2. List of differentially methylated probes (DMPs) in metabolic syndrome (MetS) compared to levels in control

Table S3. List of functionally enriched genes with hyper/hypomethylation in metabolic syndrome (MetS) compared to control levels.

Table S4. List of differentially expressed genes (DEGs) in metabolic syndrome (MetS) compared to control levels.

Table S5. List of functionally enriched differentially expressed genes (DEGs) using DisGeNET gene sets.

Table S6. List of significantly correlated genes between DNA methylation and gene expression.

Table S7. List of integrated functional network genes and pathways.

## 2 Experimental Design, Materials, and Methods

To obtain these data, 32 participants were enrolled. We conducted genome-wide DNA methylation profiling using the Infinium MethylationEPIC BeadChIP Kit (Illumina, San Diego, CA) and characterized the whole transcriptome via RNA sequencing for patients with MetS (n = 11) and controls (n = 9). The participant characteristics are shown in Table S1.

### 2.1 Subjects

Diagnosis of MetS was based on the National Cholesterol Education Program-Adult Treatment Panel (NCEP-ATP) III [2] criteria presented by the American Heart Association/National Heart, Lung, and Blood Institute. The diagnostic criteria for MetS risk factors included (1) high blood pressure (systolic blood pressure ≥ 130 mmHg, diastolic blood pressure ≥ 85 mmHg) or specific treatment, (2) fasting plasma glucose ≥ 100 mg/dL or specific treatment, (3) low HDL cholesterol (HDL-C < 40 mg/dL for men, HDL-C < 50 mg/dL for women) or specific treatment, (4) high triglyceride levels (150 mg/dL) or specific treatment, and (5) abdominal obesity with cutoffs specific to South Koreans (waist circumference ≥ 90 cm for men or ≥ 85 cm for women). MetS was defined when three or more of the aforementioned five risk factors were applicable.

### 2.2 DNA/RNA extraction

Genomic DNA and total RNA from peripheral blood mononuclear cells (PBMCs) were isolated using the AllPrep DNA/RNA Mini Kit (Qiagen, Hilden, Germany) according to the manufacturer's protocol. RNA quality was evaluated using an Agilent RNA Nano Kit (Agilent Technologies, Santa Clara, CA). Samples with RNA integrity number values > 7 were used for library construction.

### 2.3 DNA methylation data production and analysis

Bisulfite-converted genomic DNA from the samples was hybridized to the Illumina Infinium MethylationEPIC BeadChIP array (Illumina). The Illumina iScan scanner was used to scan the BeadChIP array. The raw intensity data were imported into R v. 4.0.3. The R package ChAMP v. 2.20.1 [3] was used for data preprocessing and normalization and to compare the MetS and control groups. Probe-wise differential methylation analysis was performed using ChAMP. The threshold for statistical significance of DMPs was a Benjamini-Hochberg adjusted *P* < 0.05. We used the R package EnrichR [4] to determine DMP pathway enrichment. All array data processing and analyses were conducted in R v. 4.0.3.

### 2.4 RNA-seq data production and analysis

The library was prepared using the MGIEasy RNA Directional Library Prep Kit (MGI-Tech, Shenzhen, China) and sequenced using an MGISEQ-2000 platform, to generate 100 bp paired-end reads. Reads were trimmed using Skewer v. 2.2.1 to remove adapter sequences and reads with low sequence quality. High-quality sequence reads were mapped to the hg38 human genome (UCSC Genome Browser, https://genome.ucsc.edu/) using STAR v. 2.5 [5]. mRNA expression levels were quantified using Cuffquant in Cufflinks v. 2.2.1 [6] with the “–library-type” setting “fr-firststrand”. Differential expression between the MetS and control groups was analyzed using the R package edgeR [7]. The criteria for differential expression were |log(fold-change)| > 2 and *P* < 0.05. We used EnrichR for DEG pathway enrichment analysis.

### 2.5 Combined analysis of DNA methylation and gene expression

To calculate correlation values between DNA methylation and corresponding gene expression, we first annotated the DMPs closest to the target gene according to the MethylationEPIC array annotation file. Correlations were subsequently measured via Pearson's correlation coefficient using R software (v.3.6.0).

## Ethics Statements

Informed consent was obtained from all participants in the associated study. The Institutional Review Board at the Dunsan Korean Medicine Hospital of Daejeon University reviewed and approved this study (IRB No. DJDSKH-17-BM-12). All procedures involving human participants were in accordance with the ethical standards of the institutional and/or national research committee and with the 1964 Helsinki declaration and its later amendments or comparable ethical standards.

## CRediT Author Statement

**Su-Jin Baek:** Data analysis and writing-editing manuscript; **Siwoo Lee:** conceptualization and review; **Hee-Jeong Jin:** supervision and writing of the manuscript.

## Declaration of Competing Interest

The authors declare that they have no known competing financial interests or personal relationships that could have appeared to influence the work reported in this paper.

## Data Availability

MethylationEPIC BeadChIP and RNA-seq data (Reference data) (GEO).
